# Prognostic impact of clinical course-specific mRNA expression profiles in the serum of perioperative patients with esophageal cancer in the ICU: a case control study

**DOI:** 10.1186/1479-5876-8-103

**Published:** 2010-10-22

**Authors:** Shunsaku Takahashi, Norimasa Miura, Tomomi Harada, ZhongZhi Wang, Xinhui Wang, Hideyuki Tsubokura, Yoshiaki Oshima, Junichi Hasegawa, Yoshimi Inagaki, Goshi Shiota

**Affiliations:** 1 Division of Anesthesiology and Critical Care Medicine, Tottori University School of Medicine, Nishicho 36-1, Yonago, Tottori 683-8503, Japan; 2 Division of Pharmacotherapeutics, Department of Pathophysiological and Therapeutic Science, Faculty of Medicine, Tottori University, Nishicho 86, Yonago, Tottori 683-8503, Japan; 3 Division of Anesthesiology, Tottori Red Cross Hospital, 117 Shoutokucho, Tottori, Tottori 680-8517, Japan; 4 Division of Anesthesiology, Shimane Prefectural Central Hospital, 4-1-1 Himehara, Izumo, Shimane 693-8555, Japan; 5 Division of Molecular and Genetic Medicine, Department of Genetic Medicine and Regenerative Therapeutics, Tottori University, Nishicho 86, Yonago, Tottori 683-8503, Japan

## Abstract

**Background:**

We previously reported that measuring circulating serum mRNAs using quantitative one-step real-time RT-PCR was clinically useful for detecting malignancies and determining prognosis. The aim of our study was to find crucial serum mRNA biomarkers in esophageal cancer that would provide prognostic information for post-esophagectomy patients in the critical care setting.

**Methods:**

We measured serum mRNA levels of 11 inflammatory-related genes in 27 post-esophagectomy patients admitted to the intensive care unit (ICU). We tracked these levels chronologically, perioperatively and postoperatively, until the two-week mark, investigating their clinical and prognostic significance as compared with clinical parameters. Furthermore, we investigated whether gene expression can accurately predict clinical outcome and prognosis.

**Results:**

Circulating mRNAs in postoperative esophagectomy patients had gene-specific expression profiles that varied with the clinical phase of their treatment. Multivariate regression analysis showed that upregulation of IL-6, VWF and TGF-β1 mRNA in the intraoperative phase (p = 0.016, 0.0021 and 0.009) and NAMPT and MUC1 mRNA on postoperative day 3 (p < 0.01) were independent factors of mortality in the first year of follow-up. Duration of ventilator dependence (DVD) and ICU stay were independent factors of poor prognosis (p < 0.05). Therapeutic use of Sivelestat (Elaspol^®^, Ono Pharmaceutical Co., Ltd.) significantly correlated with MUC1 and NAMPT mRNA expression (p = 0.048 and 0.045). IL-6 mRNA correlated with hypercytokinemia and recovery from hypercytokinemia (sensitivity 80.9%) and was a significant biomarker in predicting the onset of severe inflammatory diseases.

**Conclusion:**

Chronological tracking of postoperative mRNA levels of inflammatory-related genes in esophageal cancer patients may facilitate early institution of pharamacologic therapy, prediction of treatment response, and prognostication during ICU management in the perioperative period.

## Background

Esophageal cancer is one of the most aggressive malignant tumors of the digestive tract. Post-esophagectomy anastomotic leak and pneumonia are common and can lead to acute respiratory distress syndrome (ARDS). Acute respiratory distress syndrome (ARDS) is a diffuse heterogeneous lung disease resulting in progressive hypoxemia due to ventilation/perfusion mismatching and intrapulmonary shunting. Its causes are diverse and it is associated with a near 100% mortality after 48 hours [1,2]. Ventilator-induced acute lung injury (ALI) is known to cause diffuse parenchymal damage secondary to alveolar overdistension, bacterial translocation and cytokine release [3,4]. Detailed, sequential assessment of organ dysfunction during the first 48 hours of ICU admission is a reliable indicator of prognosis [5].

Recently, the use of gene-expression profiling on a transcriptome level of peripheral blood mononuclear cells (PBMC) identifies signature genes that distinguish severe sepsis (SS) from noninfectious causes of systemic inflammatory response syndrome (SIRS), sepsis-related immunosuppression and reduced inflammatory response [6]. SS has been categorized as a subset of SIRS resulting from hypercytokinemia [7]. As there are currently no reliable genetic markers for use in ICU care and prognostication, we aimed to determine the clinical value of measuring circulating RNA in the serum of ICU patients [8]. Since circulating RNA remains stable for approximately 24 hours, its detection may reflect early changes in clinical status and may make it possible to predict morbidity and survival [9].

We previously reported that the measurement of human telomerase reverse transcriptase gene (hTERT) mRNA in serum is useful for the diagnosis of some malignancies. We also found that serum transforming growth factor-α mRNA is useful as a prognostic indicator in fulminant hepatitis in patients without encephalopathy upon admission [10,11]. In the present study, we examined 11 proinflammatory genes in patients receiving therapy in the ICU following surgery for esophageal cancer: matrix metallopeptidase 9 (MMP9), which reflects the activity of neutrophils and correlates with survival in patients with esophageal cancer [12-14]; early growth response 1 (EGR1), as a transcriptional regulator in ALI [15-17]; high-mobility group box 1 (HMGB1), as a candidate proinflammatory factor predicting the prognosis of SIRS [18-20]; mucin1 (MUC1), as both an independent predictor for intravascular coagulation in ARDS and a biomarker for esophageal cancer [21-23]; nicotinamide phosphoribosyltransferase (NAMPT/PBEF1), as a regulator in new inflammatory networks [24-27]; platelet-derived growth factor alpha polypeptide (PDGFA), which is involved in alveolar septal formation [28-30]; transforming growth factor beta 1 (TGF-β1), as an activator of procollagen I in patients with acute lung injury (ALI) [31-33]; tumor necrosis factor-alpha (TNF-α), as a prognostic determinant of ARDS in adults [34-36]; von Willebrand factor (VWF), as an independent marker of poor outcome in patients with early ALI [37-39]; and interleukin 6 (IL-6), which is upregulated in inflammation and promotes the maturation of B cells [40]. Lung injury-related genes (HMGB1, MUC1 and VWF), proinflammation-related genes (MMP, CRP, and HMGB1), coagulation-related genes, immunoreactive genes (PBEF1 and TNF-α), fibrosis-related gene (TGF-β), wound-healing related gene (PDGFA), and cancer-related genes (MUC1 and hTERT) have been reported previously to correlate with the onset of ARDS or SIRS and subsequent survival. ARDS and SIRS seriously affect the prognosis of postoperative patients. Anastomotic leak and pneumonia extend the length of ICU stay and duration of ventilator dependence, resulting in a poorer prognosis. We investigated the clinical significance and prognostic usefulness of measuring serum levels of mRNA of these genes chronologically from ICU admission in patients treated surgically for esophageal cancer.

## Methods

### Patients and sample collection

27 patients who underwent radical surgery for esophageal cancer at Tottori University Hospital, Tottori Red Cross Hospital and Shimane Prefectural Central Hospital, between January 2006 and December 2008, were prospectively studied (Tables 1, 2). All patients were admitted to the ICU after operation as per our department **/**Tottori University protocol. The patients were discharged from the ICU when stable according our critical care departmental criteria.

**Table 1:** Patient Demographics in ICU After Surgical Treatment of Esophageal Cancer

Pt.#	*Con/Siv	DVD	ICU stay	Operation time	Anesthesia time	**PaO2/FiO2 ratio **(**POD1**)	SIRS	**SOFA scores **(**POD1**)	Anastomotic Leak	Pneumonia	**Mortality **(**-30D**)	**Mortality **(**-6M**)	**Mortality **(**-1Y**)
#1	Siv	2	3	765	856	211.3	2	3	+(POD8)	-	alive	alive	dead
#2	Siv	2	2	510	560	272.5	6	5	-	+(POD3)	alive	alive	dead
#3	Con	0	2	270	343	125	1	4	-	-	alive	alive	alive
#4	Siv	2	2	555	638	148.8	0	3	+(POD5)	-	alive	alive	alive
#5	Con	2	6	233	370	220	0	7	-	-	alive	alive	alive
#6	Con	7	6	930	1025	302.5	4	5	+(POD9)	+(POD7)	alive	alive	alive
#7	Siv	2	3	525	565	148	2	4	+(POD5)	-	alive	alive	alive
#8	Con	0	4	285	400	246	1	2	-	-	alive	alive	alive
#9	Siv	2	12	467	580	206	5	5	+(POD6)	-	alive	alive	alive
#10	Con	2	3	681	573	194.3	8	5	+(POD5)	-	alive	alive	dead
#11	Siv	74	74	615	682	190	31	6	+(POD5)	+(POD2)	alive	dead	dead
#12	Con	2	3	491	598	184	2	6	-	-	alive	alive	alive
#13	Con	2	3	487	570	212	1	3	+(POD5)	-	alive	alive	alive
#14	Con	6	7	543	630	447.5	7	2	-	+(POD3)	alive	alive	alive
#15	Siv	0	0	415	505	213.8	3	2	-	-	alive	alive	alive
#16	Siv	3	16	551	695	272.2	1	6	+(POD5)	-	dead	dead	dead
#17	Con	2	3	645	715	244	7	5	+(POD7)	-	alive	alive	alive
#18	Siv	11	13	547	602	277.5	2	4	-	-	alive	alive	alive
#19	Siv	3	4	520	564	342.5	1	4	-	-	alive	alive	alive
#20	Siv	3	4	698	775	397.5	1	6	+(POD12)	+(POD3)	alive	alive	alive
#21	Con	6	7	657	760	360	3	4	-	+(POD6)	alive	alive	alive
#22	Siv	4	6	1305	1375	187.5	1	9	-	-	alive	alive	alive
#23	Con	2	3	735	820	257	5	2	-	+(POD7)	alive	alive	alive
#24	Con	3	8	724	795	252	2	5	-	-	alive	alive	alive
#25	Con	1	5	525	580	216	1	2	+(POD5)	-	alive	alive	alive
#26	Con	5	7	254	345	245	0	5	-	-	alive	alive	alive
#27	Siv	3	8	572	629	257.5	5	6	-	+(POD4)	alive	alive	alive

Pt #: patient number, * con: conventional therapy, Siv: Sivelestat. DVD; duration of ventilator dependence

**Table 2:** Gene Expression Data for Esophageal Cancer Patients in the ICU After POD 14

Pt.#	SCC (ng/mL)	Cytokeratin fragment 19 (ng/mL)	CEA (ng/mL)	hTERTmRNA (logarithmic copy number)	Recurrence	Depth of tumor invasion
#1	2.4	2.5	-	3.31	-	Mp
#2	-	-	-	3.9	-	Sm
#3	0.9	1.5	-	2.96	+	Ss
#4	< 0.5	1.4	-	4.41	-	Sm
#5	-	-	-	0	-	M
#6	2.0	1.6	-	0	+	Ss
#7	0.9	0.9	-	4.96	-	Sm
#8	< 0.5	1.9	-	0	-	Ss
#9	1.1	1.3	3.8	2.97	-	Ss
#10	1.2	2.1	1.0	3.58	-	Ss
#11	-	-	-	2.75	-	Ss
#12	0.8	0.9	-	2.14	-	Ss
#13	1.6	2.7	1.4	4.61	-	Sm
#14	2.7	-	3.1	3.99	-	Ss
#15	0.9	3.0	-	3.84	-	Ss
#16	-	-	-	3.81	-	Sm
#17	1.6	7.4	-	4.14	-	Mp
#18	1.3	0.9	2.4	4.53	-	Sm
#19	0.7	2.5	-	4.11	-	Sm
#20	3.9	3.1	2.4	4.41	-	Ss
#21	2.9	-	3.5	3.44	-	Mp
#22	0.7	2.2	-	4.35	-	Sm
#23	< 0.5	1.4	-	4.03	-	Ss
#24	1.4	-	-	3.89	-	M
#25	1.2	1.4	-	3.35	-	Ss
#26	-	-	-	4.39	-	M
#27	0.7	0.9	2.3	4.45	-	Mp

-: not measured

We measured serum mRNA levels for 14 days postoperatively. Informed consent was obtained from each patient and study protocols followed standard ethical guidelines (Declaration of Helsinki, 1975) and were approved by the institutional review board of Tottori University (approval no.138, no 138 1, 2001; no. 343, 2009). The patients consisted of 3 females (mean age 67.3 years, age range 49 to 82 years) and 24 males (mean age 65 years, age range 40-76). All patients were classified as American Society of Anesthesiologists (ASA) physical status 1 or 2. Patients were prospectively followed for 12 months postoperatively. SIRS or ARDS were diagnosed according to accepted consensus definitions [41,42]. Clinicopathological findings, such as age, diagnosis, etiology, prognosis, effect of the neutrophil elastase inhibitor sivelestat (4.8 mg/kg/day), total days of ventilator dependence (DVD), total days of ICU stay, preoperative CRP levels (preCRP), CRP levels at postoperative day (POD) 1, peak concentrations of CRP (peak CRP), operation duration, anesthesia duration, PaO_2_/FiO_2 _ratio at POD 1, days of SIRS, sequential organ failure assessment (SOFA) scores at POD 1, and mortality at 30 days, 6 months, and 1 year were recorded.

Anesthesia consisted of general anesthesia and epidural anesthesia. After surgery, all patients were reintubated with single-lumen endotracheal tubes from the double-lumen endotracheal tubes used intraoperatively and received ventilator support in ICU. Serum from whole blood was obtained intraoperatively and on POD 1, POD 3, POD 5 and POD 14. We measured serum mRNA levels of 11 genes (MMP9, CRP, HMGB1, MUC1, EGR1, PBEF1, PDGFA, TGF-β1, TNF-α, VWF, and IL-6). Sivelestat was prophylactically administered intravenously by the judgment of the attending physician and according to the manufacturer's recommendations. We distinguished SIRS from severe non-infectious systemic inflammatory response syndrome (SNISIRS) by examining gene expression (GE) in the serum and synchronizing GE changes with the clinical course of events.

Processing of the blood and serum samples was performed after blood sampling during the operation and at POD 1, POD 3, POD 5 and POD 14. mRNA quantification was performed as previously described [43]. RNA extraction and real-time RT-PCR RNA was performed after DNase treatment, also reported previously [43-45]. In brief, RNA from 200 μl of serum was dissolved in 200 μl of H_2_O. RT-PCR was performed using 1 μl of RNA extract and 2 μl of SYBR Green I (Roche, Basel, Switzerland) in a one-step RT-PCR kit (Qiagen, Tokyo, Japan). RNA was extracted from blood using the same volume of serum concentrated 20-fold (Invitrogen Corp., Carlsbad, CA, USA). RT-PCR conditions were: incubation at 50°C for 30 min followed by incubation for 12 min at 95°C for denaturation, then 50 cycles at 95°C (0 s), annealing at 50-55°C (10 s) and 72°C (15 s), and extension at 40°C (20 s). All primers were optimally designed (INTEC Web & Genome Informatics Corp., Tokyo, Japan). The final concentration of the primers was 1 μM; sequences are shown in Table 3. The dynamic range of the real-time PCR analysis for each mRNA was more than 5-10 copies in this assay, but we semi-quantitatively measured 11 gene expression profiles of interest as relative expression levels against β-actin mRNA [46]. The RT-PCR assay was repeated twice and quantification was reproducibly confirmed with LineGene (TOYOBO, Tokyo, Japan). We confirmed that the amplicons were derived from the gene of interest by Western blot. IL-6 protein level was measured using an ELISA kit according to the manufacturer's instruction (R&D Systems, MN, USA). SOFA was scored according to international criteria [47].

**Table 3:** Primer Sets Used for Each Gene Investigated

Gene	**GenBank Accession No**.	Forward Primer	Reverse Primer
VWF	NM_000552.2	TGA CCA GGT TCT CCG AGG AG	CAC ACG TCG TAG CGG CAG TT
TGFB1	MN_000660.3	GAC TAC TAC GCC AAG GAG GT	GGA GCT CTG ATG TGT TGA AG
PDGFA	MN_002607.5	GGG AGT GAG GAT TCT TTG GA	AAA TGA CCG TCC TGG TCT TT
NAMPT	MN_005746.2	CTG TTC CTG AGG GCA TTG TC	GGC CAC TGT GAT TGG ATA CC
CRP	MN_000567.2	ACA GTG GGT GGG TCT GAA AT	TAC CCA GAA CTC CAC GAT CC
EGR1	MN_001964.2	TTC TTC GTC CTT TTG GTT TA	CTT AAG GCT AGA GGT GAG CA
HMBG1	MN_002128.4	AAC CAC CCA GAT GCT TCA GT	TCC GCT TTT GCC ATA TCT TC
TNF-α	MN_000594.2	TGC TTG TTC CTC AGC CTC TT	GCA CTC ACC TCT TCC CTC TG
MUC1	MN_001018016.1	CCA TTC CAC TCC ACT CAG GT	CCT CTG AAG GAG GCT GTG AT
MMP9	NM_004994.2	TTG ACA GCG ACA AGA AGT GG	CCC TCA GTC AAG CGC TAC AT
IL-6	MN_000600.3	ATG CAA TAA CCA CCC CTG AC	TAA AGC TGC GCA CAA TGA GA
β-actin	MN_031144.2	ACC TGA CTG ACT ACC TCA TG	GCA GCC GTG GCC ATC TCT TG

### Statistical Analysis

Clinical parameters and gene expression profiles were statistically evaluated using SPSS 13.0 (SPSS Japan Inc., an IBM company, 2004). Multivariate regression analysis was performed with respect to prognosis weighted at 30 days, 6 months and 12 months or with stepwise selection. In addition, we tested the effects of sivelestat, polymyxin B-immobilized fiber column (PMX), and factors predicting the development anastomotic leak or pneumonia against clinical course and gene expression by one-way ANOVA. To assess the accuracy of the prognostic factors (medication use, gene function in the acute phase, and ventilatory regulation), the correlation of each factor with prognosis was evaluated using receiver operator characteristic (ROC) curve analyses. A predictive cut-off value was evaluated as the nearest point from the left upper edge of the ROC curve analysis graph. Sensitivity was calculated as the mean confidence interval (CI) of the area under the curve (AUC) and specificity was calculated in the output table of the ROC. To estimate survival, Kaplan-Meier analysis was performed. P values less than 0.05 were considered statistically significant.

## Results

### Circulating mRNA expression during hospitalization

**Figure 1:**
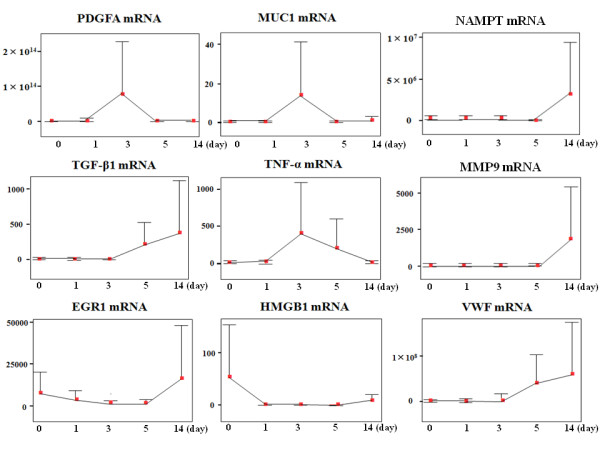
**Each mRNA expression profiles during 14 days at ICU**. Changes in the circulating mRNA expression profile during the clinical course (post-operative days [POD] 0-14) in ICU. Relative ratio of mRNA expression compared with β-actin mRNA in serum is depicted as the longitudinal axis. We show the change in mRNA expression level for PDGFA, MUC1, PBEF1/NAMPT, TGF-β1, TNF-α, MMP9, EGR1, HMGB1, and VWF. IL-6 data and CRP data are provided in Figure 3 and Additional file 1, respectively. HMGB1 and EGR1 responded to surgery and being upregulated at POD 0. PDGFA, MUC1, and TNF-α peaked at POD 3. TGF-β1 and VWF started being upregulated from POD 3. PBEF1/NAMPT and MMP9 started being upregulated from POD 5. All genes examined in this study were upregulated at equal or greater levels than the level of β-actin mRNA during the 14 days of ICU stay.

The mRNA expression profiles over the 14 days are shown in Figure 1. MMP9 and NAMPT (PBEF1) were similar in that both were upregulated from POD 5 onwards. VWF and TGF-β1 demonstrated similar upregulation from POD 3. At POD 1, CRP mRNA upregulation was accompanied by increased serum CRP levels; these decreased at POD 3 following appropriate treatment (data not shown). MUC1 and PDGFA were upregulated at POD 3 (p = 0.048 and 0.045), followed by recovery from POD 5 to POD 14. IL-6 was upregulated at POD 5 then decreased to the intraoperative baseline value. EGR1 and HMGB1 levels gradually decreased from the intraoperative values to baseline at POD 14.

**Figure 2:**
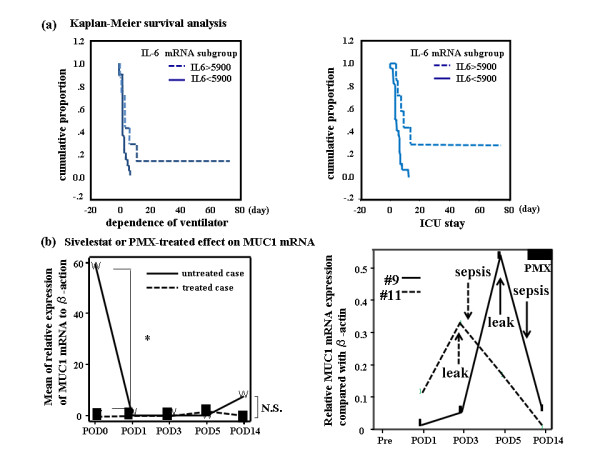
**Correlation between IL-6 mRNA expression and clinical parameters (panel a) and effects of Sivelestat or PMX-treatment on MUC1 mRNA (panel b)**. (a) (left) (a) Kaplan-Meier plot for two conditions (IL-6 mRNA during operation is classified as categorized more or less than 5900) associated with the clinical course of the patients. If the IL-6 mRNA was downregulated to < 5900, the cumulative proportion had a tendency to increase (p = 0.0505), resulting in a shorter period of ventilator dependence. Solid line and dotted line refer to < 5900 and > 5900, respectively. (right) If IL-6 mRNA was downregulated to < 5900, the cumulative proportion was improved significantly (p = 0.0062), resulting in a shorter ICU stay. (b) To describe the therapeutic effects of gene expression on prognosis, the effect on MUC1 mRNA of treatment with sivelestat- (left) or a polymyxin B-immobilized fiber column (PMX) (right) are depicted. Dotted line and solid line refer to sivelestat-treated (n = 13) and untreated (n = 14) patients, respectively. In both cases, the mean value of MUC1 mRNA expression relative to β-action mRNA is shown. (left) Sivelestat caused a significant change in the genes of interest (Table 5). However, it had no significant influence on prognosis (recovery from SIRS). *: p < 0.05, N.S.: not significant. (right) Clinical courses in two PMX-treated cases (#9: solid line and #11: dotted line) are shown. There were no significant relationships between any of the genes and the therapeutic modalities required. Relative MUC1 mRNA expression compared with β-actin mRNA is plotted.

Two mRNAs of proinflammatory genes seen in ALI (HMGB1 and VWF) changed similarly (p = 0.021). CRP mRNA correlated with conventional CRP levels (p = 0.029 and 0.004). Primers designed for amplifying CRP mRNA did not detect inflammation with more sensitivity than conventional CRP. However, CRP mRNA correlated with CRP levels at PODs 1, 3, and 14 (p = 0.009, 0.02, and 0.009). Sensitivities and specificities of mRNA levels as prognostic indicators of clinical course are shown (Additional File 1). With respect to gene markers' association with surgical parameters, upregulation of TNF-α mRNA correlated with increased duration of anesthesia (p = 0.023); and VWF upregulation with increased duration of surgery (p = 0.025). MMP9 mRNA expression correlated with PDGFA mRNA up to POD 14 (p = 0.007) and treatment with sivelestat altered MUC1 expression (p = 0.024, Figure 2b). TNF-α mRNA expression correlated with duration of SIRS (p = 0.042). CRP mRNA expression correlated with length of ICU stay, which in turn was associated with 6-month mortality (p = 0.033 and 0.016).

### Prognostic factors in the perioperative period

**Figure 3:**
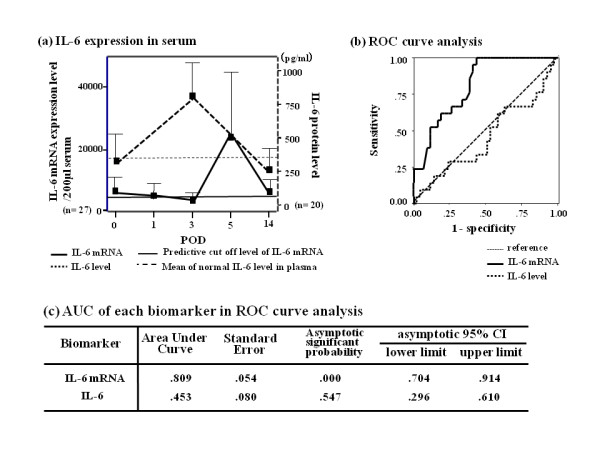
**IL-6 mRNA expression and IL-6 protein level**. IL-6 mRNA expression and the IL-6 protein level were evaluated using expression profiles and receiver operating characteristic (ROC) curve analysis. (a) Transcriptional (n = 27) and translational (n = 20) profiles of IL-6 in serum are shown from POD 0 to 14 (CI: 95%), based on the relative expression ratio compared with β-actin mRNA. Bold solid line, bold dotted line, solid line, and dotted line depict IL-6 mRNA, IL-6, predictive cut-off level of IL-6 mRNA and mean of normal IL-6 level in plasma, respectively. (b) ROC curve analysis drawn between IL-6 mRNA and IL-6. Bold solid line, bold dotted line, dotted line, and solid line refer to IL-6 mRNA, IL-6, mean of normal IL-6 level in plasma, and predictive cut-off level of IL-6 mRNA, respectively. (c) SPSS software analysis of the AUC of the ROC curve was 0.809 and 0.453 for IL-6 mRNA and IL-6, respectively.

We found IL-6 mRNA to be a significant marker of prognosis (Figure 3b, c). IL-6 mRNA was upregulated in the immediate perioperative period (POD 0, Figure 3a) and gradually decreased at POD 3. Conversely, IL-6 levels increased postoperatively. The AUC of IL-6 mRNA and IL-6 was 0.809 and 0.453, respectively, and the predictive cut-off value of IL-6 mRNA was 3400 as a relative ratio to the β-actin copy number.

The stepwise analysis is shown in Table 4, suggesting that a high level of IL-6 mRNA at POD 0 is an independent indicator of poor prognosis (as are days of ventilator dependence, days of ICU stay, and days of SIRS (p < 0.0001); a high level at POD3 predicted the onset of pneumonia (p = 0.021). Days of ventilator dependence, days of ICU stay, and SIRS days were independent factors influencing prognosis (p < 0.05; data not shown). A significant reduction in mortality was seen by gene expression changes on POD 14 (p < 0.001 by one-way ANOVA). Upregulation of VWF and TGF-ß1 mRNA intraoperatively correlated with mortality (p = 0.0021 and 0.009). POD 1 upregulation of PDGFA, ERG1, and HMGB1 mRNA correlated significantly with worse prognosis. (p = 0.009, 0.004, and 0.012). At POD 3, NAMPT and MUC1 mRNA were found to be independent prognostic factors for 1-year mortality (p = 0.007, 0.012); at POD 14, NAMPT mRNA correlated with mortality at 30 days and 1 year (p < 0.0001 and p = 0.0016).

**Table 4:** Logistic Regression Analysis of Morbidity and Mortality With Stepwise Selection

	gene	p value	R		gene	p value	R		gene	p value	R
**DVD**				**1Y-mortality**				**6M-mortality**			
POD0	IL-6	< 0.0001	0.787	POD0	IL-6	0.016	0.69	POD0	VWF	< 0.0001	0.893
**days of ICU stay**					VWF	0.021			IL-6	< 0.0001	
POD0	IL-6	< 0.0001	0.813		TGF-β1	0.009		**30D-mortality**			
**days of SIRS**				POD1	PDGFA	0.009	0.71	POD0	VWF	< 0.0001	1.000
POD0	IL-6	< 0.0001	0.738		ERG1	0.004			NAMPT	< 0.0001	
**Pneumonia**					HMGB1	0.012		POD5	HMGB1	< 0.0001	0.993
POD3	IL-6	0.021	0.442	POD3	NAMPT	0.007	0.63		MUC1	0.002	
**PaO**_**2**_**/FiO**_**2 **_**ratio**					MUC1	0.012		POD14	NAMPT	< 0.0001	1.000
POD1	MMP9	0.034	0.409	POD14	ERG1	0.0016	0.59		PDGFA	< 0.0001	
**SOFA score**					NAMPT	0.0016			CRP	0.015	
POD1	TGF-β1	0.005	0.528								

N.A: no applicable; N.S.: not significant. R: correlation coefficient.

DVD: duration of ventilator dependence. Independence variables are MMP9, CRP (pre, POD1, peak), ERG, HMGB1, MUC1, PBEF, PDGFA, TGF-β1, TNF-α, VWF, IL-6, Sivelestat, PMX, anesthesia and operation time.

Sivelestat affected suppressive gene expression of CRP, EGR1, MUC1, TNF-α, PDGFA, NAMPT, and VWF (Table 5). However, PMX treatment did not improve clinical outcome (Figure 2b). The SOFA score correlated only with days of ventilator dependence and ICU stay (p = 0.038 and 0.039, Additional File 2).

**Table 5:** One-Way ANOVA Analysis With Respect to Sivelestat

Time course	Genetic parameters	P value	Contribution to prognosis	P value
POD 1	EGR1 mRNA	0.037	30D-mortality	0.032
	MUC1 mRNA	0.041	6M-mortality	0.001
	PDGFA mRNA	0.037		
	TNF-α mRNA	0.016		
	VWF mRNA	0.033		
POD 3	CRP mRNA	0.023	6M-mortality	< 0.001
	EGR1 mRNA	0.022	1Y-mortality	0.023
	MUC1 mRNA	0.048		
	NAMPT mRNA	0.045		
	PDGFA mRNA	0.032		
	TGF-β1 mRNA	0.016		
	TNF-α mRNA	0.020		
	VWF mRNA	0.047		
POD 5	CRP mRNA	0.001	30D-mortality	0.032
	TNF-α mRNA	0.032	6M-mortality	0.001
POD 14	MMP9 mRNA	0.047	30D-mortality	0.032
	EGR1 mRNA	0.034	6M-mortality	0.001
	HMGB1 mRNA	0.042		
	NAMPT mRNA	0.032		
	TGF-β1 mRNA	0.048		
	VWF mRNA	0.032		

12/27 (44%) patients experienced anastomotic leak (9 cervical and 3 thoracic, additional file 3). EGR1 and IL-6 mRNA expression correlated with anastomotic leak and pneumonia at POD 3 by regression analysis (p = 0.021, Table 4). Furthermore, increased duration of operation, anesthesia, and mechanical ventilation was associated with increased risk of pneumonia (p < 0.001, 0.028, and 0.022, Additional File 2, 4). PaO_2_/FiO_2 _ratio did not correlate with any other gene expressions.

## Discussion

Esophageal cancer is one of the most aggressive malignant tumors of the digestive tract. Post-esophagectomy anastomotic leak and pneumonia are common; furthermore, they prolong ICU stay and contribute to poor prognosis [48]. It is of paramount importance to diagnose these complications immediately postoperatively, and treat them expeditiously [49].

We investigated gene expression by measuring circulating ribonucleic acids in serum (CRAS), with the hope of discovering early prognostic markers post-esophagectomy. We hypothesized that the expression of certain proinflammatory genes would predict outcome, and in particular that POD 1 levels would help to identify patients at risk for anastomotic leak and pneumonia. Furthermore, we expected that gene expression on POD 14 might predict mortality.

44% (33% cervical and 11% thoracic) of our patients experienced anastomotic leak, which was greater than that which is reported in the literature (expected less than 10%) [50]. Cervical leaks were treated conservatively while thoracic leaks were severe and contributed to the high morbidity rate as described in our study. We studied the correlation between mRNA levels and morbidity and mortality. Upregulation of VWF mRNA prognosticated poor clinical condition by multivariate analysis. Upregulation of EGR and NAMPT mRNA at POD 1, 3 and 14 indicates that we should become more clinically astute in the immediate postoperative period. The mean/median/cutoff values of IL-6 mRNA are 5906/2810/5900 and, in Kaplan-Meyer survival analysis; if they did not demonstrate significant value among clinical parameters, we concluded that IL-6 mRNA was not an indicative marker for outcome. However, they did correlate with duration of ventilator dependence and ICU stay (Figure 2).

Duration of ventilator dependence, duration of ICU stay and SIRS expectedly affected 6-month mortality, independent of cancer recurrence. Since these conditions are caused by the severity of the underlying disease, by unexpected immunoreactions, and by iatrogenic lung injury in the perioperative period, interpretation of their pathogenesis is complicated. Although the onset of SIRS is critical and can adversely affect recovery, we believe that serum gene expression profiles may reliably predict prognosis because of the mRNA stability; i.e., mRNA levels directly reflect pathophysiology either in real-time or over the past 24 hours.

The changes observed in gene expression was indicative of postoperative clinical course. CRP mRNA was upregulated first, with PDGFA, TNF-α and MUC1 mRNA following by POD 3. In turn, IL-6, VWF and TGF-β1 mRNA were upregulated at POD 5, then NAMPT, EGR1 and MMP9 mRNA. Thus, gene expression actively evolves after surgery for esophageal cancer. It remains unclear whether these changes occur in other disease states.

Four patients (#11, 13, 16, 20) received long-term sivelestat and displayed significant downregulation of NAMPT and MUC1 mRNA (p < 0.001 and 0.034 by one-way ANOVA) compared with the 23 patients who did not receive this medication. Four patients (#9, 11, 16, 20) with sepsis following anastomotic leak or aspiration pneumonia significantly upregulated the same genes (p < 0.001 and p = 0.025), if statistical analysis is weighted against dead outcome. As MUC1 expression correlated with CRP, NAMPT may be a crucial factor in the pro-inflammatory state.

Microarray analysis is inefficient for detecting small amounts of circulating RNA because of the limits of current biotechniques, particularly the requirement for at least 500 ml of blood. We chose candidate genes based on information from previous reports and examined their significance. We identified VWF and TGF-β1 as potential predictors of improved prognosis, the latter being an indicator of fibrosis. VWF is a glycoprotein that binds to coagulation factor VIII. It functions as both an antihemophilic factor and a platelet-vessel wall mediator in the blood coagulation system. It is crucial to hemostasis and promotes adhesion of platelets to sites of vascular injury by forming a molecular bridge between the sub-endothelial collagen matrix and platelet-surface receptor complex GPIb-IX-V. Therefore, upregulated VWF may represent unstable hemostasis and reflect damage to endothelial megakaryocytes expressing VWF. In the signaling pathway, VWF interacts with integrins in the extracellular matrix (ECM) and has functions in the complement and coagulation cascades, linking downstream to the inflammatory process or to B cell receptor signaling.

NAMPT is another indicator for prognosis. It is the rate-limiting component in the mammalian nicotinamide adenine dinucleotide (NAD) biosynthesis pathway, and promotes vascular smooth muscle cell maturation and inhibition of neutrophil apoptosis. It was originally thought to be a cytokine that acted on early B-lineage precursor cells or T cell development, by enhancing the effect of IL-7 and SKP1-CUL1-F-box protein (SCF) on pre-B-cell colony formation. SCF mediates the ubiquitination of proteins involved in cell cycle progression, signal transduction and transcription. PDGFA is also a predictive factor for prognosis. It is activated in IFN-γ/IL-10 signaling in keratinocytes via the JAK/STAT pathway and is also involved in signaling via the MAPK cascades, STATs and NF-κB through its receptor. It contributes to balancing the Th1/Th2 switch by affecting anti-apoptosis and cell proliferation. EGR1 is targeted by Erk, is activated by IL-2 and IL-3 cascades, and targets eukaryotic translation initiation factor 4E binding protein 1. Severe inflammatory disease is a critical condition linked to collapse of the Th1/Th2 balance and, from a prognostic standpoint, these genes are upregulated when Th1 cells (producing IL-2, IL-3 and IFN-γ) are dominant over Th2 cells (generating IL-10 and leading to IL-7 activation). This suggests that novel therapeutic antibody drugs for SIRS may be found in the study of these cytokines.

TGF-α mRNA in serum has previously been described as a prognosticator in fulminant hepatitis [11] although this was not the case in our study. Expression of CRP mRNA correlated with the serum CRP level at all clinical phases, although we could not optimize the reaction condition for detecting CRP mRNA. We reconfirmed that CRP is an excellent marker for acute inflammation, but not for prognosis and SIRS onset. These prognostic genes for SIRS or sepsis may be useful in intensive care settings for earlier detection of decompensation [51].

## Conclusion

We proposed measuring an inflammatory gene expression profile perioperatively in patients undergoing surgery for esophageal cancer. VWF and TGFB1 mRNA at POD 0 were prognostic biomarkers for mortality. IL-6 mRNA was a significant biomarker for the onset of severe inflammatory conditions and its upregulation throughout the postoperative period predicted poor prognosis. We could not distinguish SIRS from bacteremia. Further prospective studies on individual gene expression profiles are necessary to clarify their influence on prognosis in esophageal cancer.

## Abbreviations

ICU: intensive care unit; MMP9: matrix metallopeptidase 9; CRP: C reactive protein; EGR1: early growth response 1; HMGB1: high-mobility group box 1; MUC1: mucin 1; NAMPT (PBEF1): nicotinamide phosphoribosyltransferase; PDGFA: platelet-derived growth factor alpha polypeptide; TGF-β1: transforming growth factor beta 1; TNF-α: tumor necrosis factor-alpha; VWF: von Willebrand factor; ARDS: acute respiratory distress syndrome; SIRS: systemic inflammatory response syndrome; SNISIRS: severe non-infectious systemic inflammatory response syndrome; GE: gene expression; SOFA score: sequential organ failure assessment score; SS: severe sepsis; DVD: duration of ventilator dependence; CRAS: circulating ribonucleic acids in serum; ALI: acute lung injury; PMX: polymyxin B-immobilized fiber column; MUC1: mucin 1; IL-6: interleukin-6; ECM: extracellular matrix; NAD: nicotinamide adenine dinucleotide; SCF: SKP1-CUL1-F.

## Competing interests

The authors declare that they have no competing interests.

## Authors' contributions

ST and MN designed experiments, interpreted data and drafted the manuscript; TH and HT managed patient samples, prepared RNA; ZW and XW performed real-time PCR; YO, JH, YI and GS provided detailed ideas and discussions.

All authors have read and approved the final manuscript.

## Supplementary Material

Additional file 1 **CRP mRNA expression and CRP protein level.** Description: Depiction of the diagnostic accuracy of CRP and mRNA levels. (a) Change in circulating CRP mRNA expression during the clinical course in ICU. Upregulation of CRP mRNA was induced at POD 1 by the surgical intervention. The longitudinal axis is relative CRP mRNA expression compared with β-actin mRNA in serum. (b) ROC curve analysis. Bold solid line, bold dotted line, and dotted line refer to CRP level, CRP mRNA and reference, respectively. (c) AUC of the ROC curve analysis of each biomarker. The sensitivities of CRP level and CRP mRNA were 98.6% and 74.1%, respectively. CRP level was superior to CRP mRNA as an inflammatory biomarker. Click here for file

Additional files 2 **Correlation between GE and clinical parameters**. To examine the relationship between clinical parameters and GE, the Pearson correlation analysis test was performed from POD 0 to POD 14. DVD: duration of ventilator dependence. Click here for file

Additional file 3 **Surgical treatment and an anastomotic leakage**. Surgical treatment and an anastomotic leakage are shown. Click here for file

Additional file 4 **Correlation between GE and clinical parameters**. To examine the relationship between clinical parameters and GE, the Pearson correlation analysis test was performed from POD 0 to POD 14. DVD: duration of ventilator dependence. Click here for file
