# Supplementary figures and images for: Prognostic impact of clinical course-specific mRNA expression profiles in the serum of perioperative patients with esophageal cancer in the ICU: a case control study
Source: J Transl Med. 2010 Oct 22;8:103. doi: 10.1186/1479-5876-8-103 (PMC2984412; doi:10.1186/1479-5876-8-103)

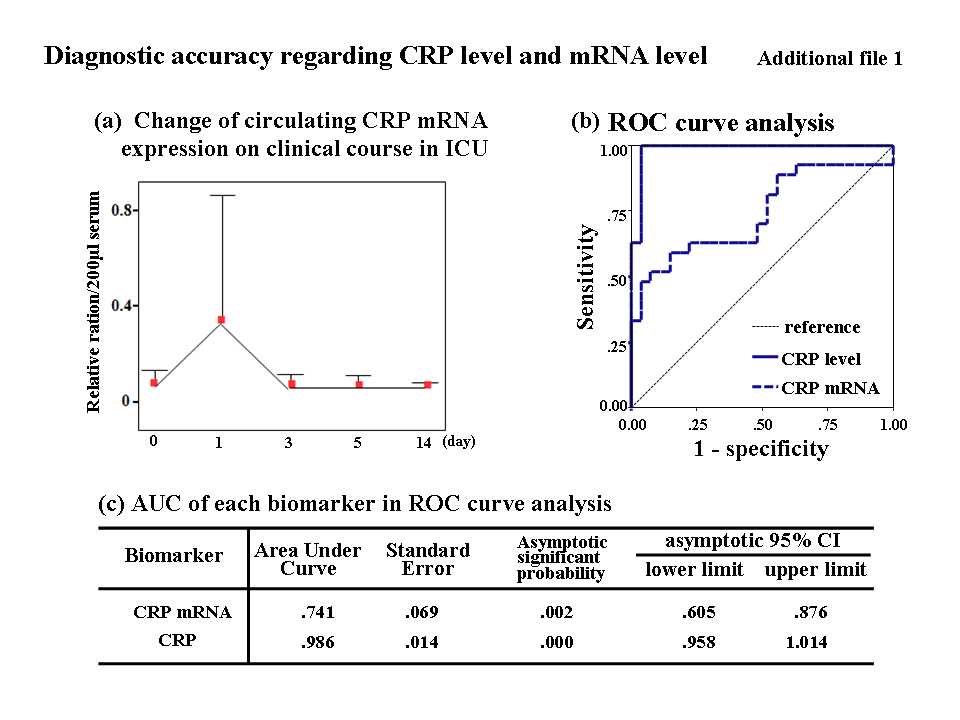

Supplement: Additional file 1 — CRP mRNA expression and CRP protein level. Description: Depiction of the diagnostic accuracy of CRP and mRNA levels. (a) Change in circulating CRP mRNA expression during the clinical course in ICU. Upregulation of CRP mRNA was induced at POD 1 by the surgical intervention. The longitudinal axis is relative CRP mRNA expression compared with β-actin mRNA in serum. (b) ROC curve analysis. Bold solid line, bold dotted line, and dotted line refer to CRP level, CRP mRNA and reference, respectively. (c) AUC of the ROC curve analysis of each biomarker. The sensitivities of CRP level and CRP mRNA were 98.6% and 74.1%, respectively. CRP level was superior to CRP mRNA as an inflammatory biomarker. [file 1479-5876-8-103-S1.TIFF]

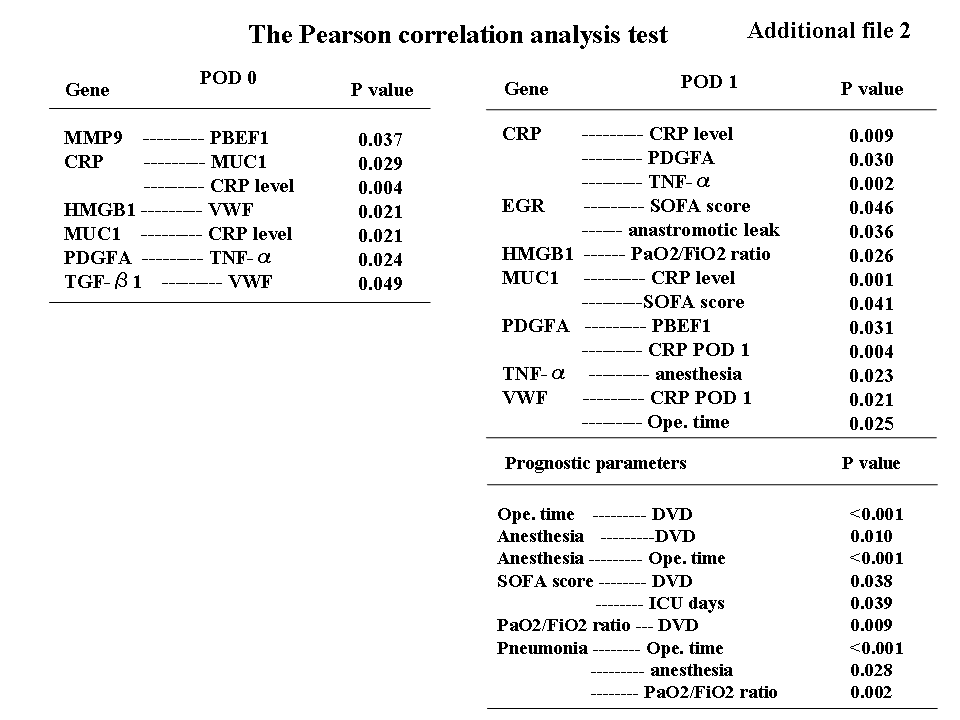

Supplement: Additional files 2 — Correlation between GE and clinical parameters. To examine the relationship between clinical parameters and GE, the Pearson correlation analysis test was performed from POD 0 to POD 14. DVD: duration of ventilator dependence. [file 1479-5876-8-103-S2.TIFF]

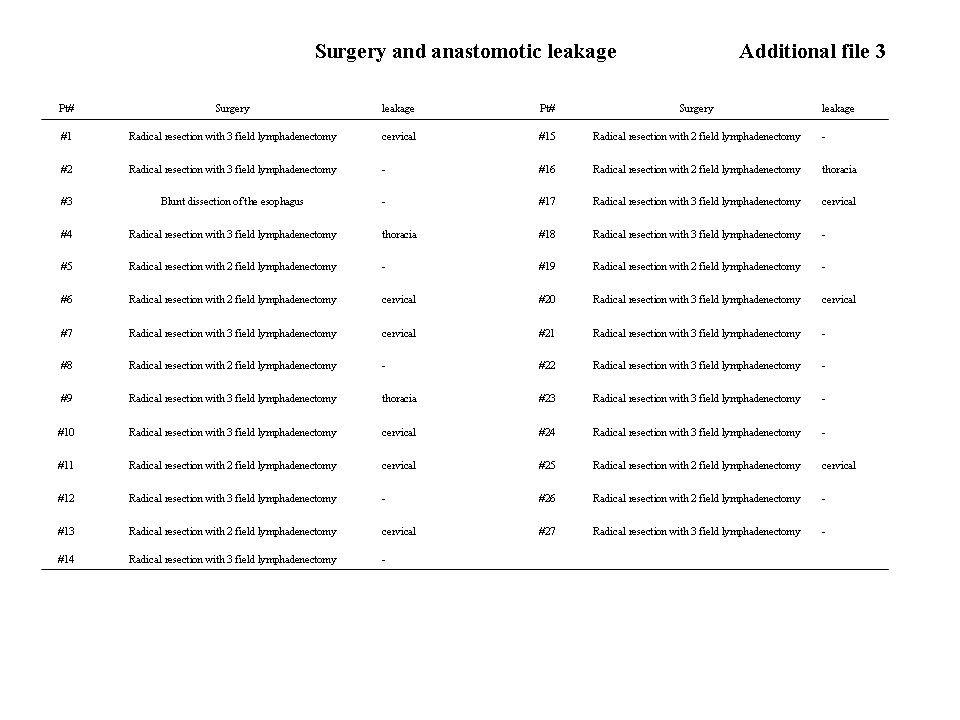

Supplement: Additional file 3 — Surgical treatment and an anastomotic leakage. Surgical treatment and an anastomotic leakage are shown. [file 1479-5876-8-103-S3.TIFF]

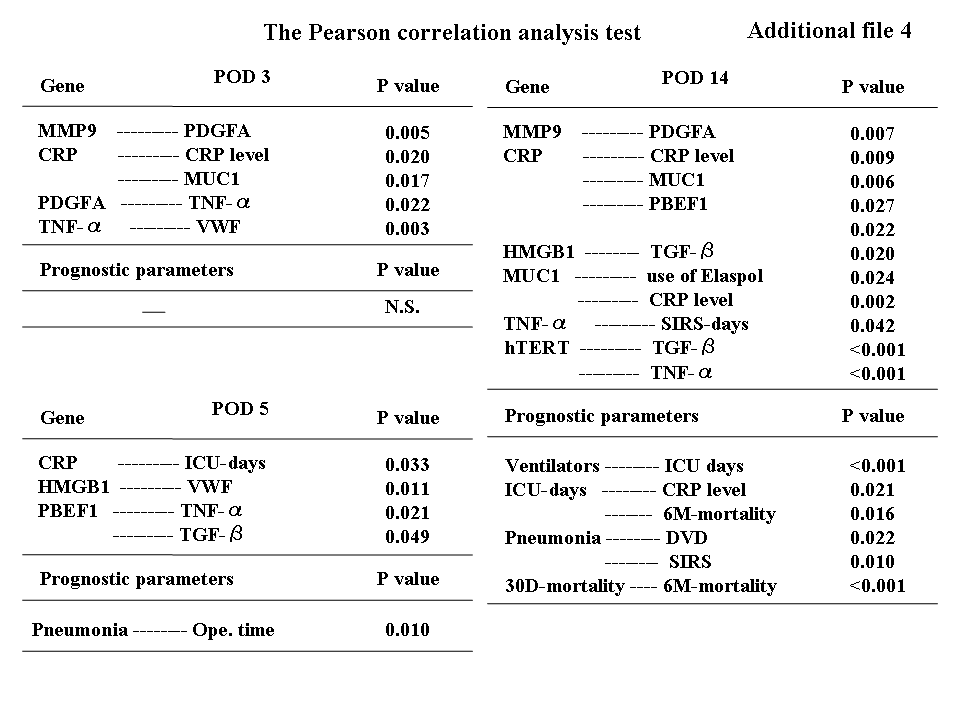

Supplement: Additional file 4 — Correlation between GE and clinical parameters. To examine the relationship between clinical parameters and GE, the Pearson correlation analysis test was performed from POD 0 to POD 14. DVD: duration of ventilator dependence. [file 1479-5876-8-103-S4.TIFF]
